# Assessing Radiosensitivity of Bladder Cancer *in vitro*: A 2D vs. 3D Approach

**DOI:** 10.3389/fonc.2019.00153

**Published:** 2019-03-19

**Authors:** Larry Bodgi, Hisham F. Bahmad, Tarek Araji, Joelle Al Choboq, Jolie Bou-Gharios, Katia Cheaito, Youssef H. Zeidan, Toufic Eid, Fady Geara, Wassim Abou-Kheir

**Affiliations:** ^1^Department of Radiation Oncology, American University of Beirut Medical Center, Beirut, Lebanon; ^2^Department of Anatomy, Cell Biology and Physiological Sciences, Faculty of Medicine, American University of Beirut, Beirut, Lebanon

**Keywords:** bladder cancer, radiosensitivity, radioresistance, cancer stem cells, modeling

## Abstract

**Background:** Bladder cancer is the fourth most commonly diagnosed cancer among males worldwide. Current treatment strategies established for bladder cancer mainly consist of cystectomy yet advances in radiation therapy have pointed to the value of organ-preserving strategies in preserving patients' quality of life.

**Aim:** To study and compare the radiosensitivity in two-dimension (2D) and physiologically-relevant three-dimension (3D) *in vitro* culture of three human bladder cancer cell lines, RT4, T24, and UM-UC-3.

**Materials and Methods:** Clonogenic assay was performed to assess cells' radiosensitivity in 2D. Employing the 3D Matrigel™-based cultures to enrich for cancer stem cells (CSCs) allowed us to assess the survival of this subpopulation of cells via evaluating the number, i.e., sphere forming unit (SFU), and the sizes of cultured spheres, formed from cells exposed to different radiation doses compared to non-irradiated cells.

**Results:** Irradiating cells with increasing radiation doses revealed highest survival rates with RT4 cells in 2D, followed by T24 and UM-UC-3. In 3D, however, UM-UC-3 cells were shown to be the most radio-resistant as evidenced by the number of spheres formed, yet they displayed the least efficient volume reduction/regression (VR), whilst the volume decreased significantly for both RT4 and T24 cells. Sphere VR and sphere ratio (SR) values were then plotted against each other demonstrating a linear correlation between volume and number with RT4 and UM-UC-3 cell lines, but not T24. Lastly, multiple regression model was employed to evaluate the possibility of obtaining a function combining both 3D parameters, SR and VR, with the surviving fraction (SF) in 2D, and showed a linear regression for T24 cells only, with a correlation coefficient of 0.97 for the combined parameters.

**Conclusion:** We were able to radiobiologically characterize 3 human bladder cancer cell lines showing differential effects of radiation between 2D and 3D culture systems, paving the way for achieving better assessment of radiosensitivity of bladder cancer *in vitro*.

## Introduction

Bladder cancer is one of the most commonly diagnosed cancers among men worldwide (1). Although cystectomy is still considered the mainstay of treatment, recent advances in radiation delivery have increased the importance of bladder preserving strategies, while also improving patients' quality of life (2–7). The introduction of radiotherapy treatments in this context highlighted the need for a radiobiological characterization of bladder cancer. Indeed, many studies have focused on the *in vitro* radio-response of bladder cancer cells. These have included DNA damage assessments, apoptosis tests, genomic analyses, and clonogenic assays; however, to date there is still no reliable bladder radiosensitivity predictive test (2, 8–11). In fact, intrinsic radiosensitivity is generally correlated with loss of clonogenicity which is directly linked to the ability of the cell to repair radiation-induced DNA damage. Specifically, DNA double-strand breaks are currently considered the key lesions responsible for radiation-induced cell death (12–14).

Out of all the radiosensitivity tests, clonogenic assay is still considered as the main reference for cells' response to ionizing radiation (IR), as it allows the quantification of radio-induced cell death (15–17). In 1981, Fertil and Malaise showed that the surviving fraction (SF) at 2 Gy can be correlated with tumor control (16). Since then, many models have been developed to describe radio-induced cell death with the linear-quadratic model still being used in daily clinical routine as it shows the best fitting quality (18–24).

On the other hand, many studies have shown that treatment failures, recurrence and metastasis can be correlated particularly to the presence of surviving subpopulation of cancer stem cells (CSCs) within tumors, that are resistant to conventional treatments (25–27). The identification of the first CSCs from acute myeloid leukemia in the haematopoietic system in 1994 (28) has given way to potential isolation of similar tissue-specific CSCs and progenitor cells from any other tumor in the body (29). Those CSCs, also referred to as tumor initiating cells, are a small subpopulation of cells residing within the tumor bulk that have similar characteristics to normal stem cells including tumor initiation, multiple differentiation, and self-renewal capabilities (30–32). It has been validated that CSCs possess the capability of forming multicellular 3D spheres *in vitro* when grown in non-adherent serum-free conditions (33–38). Such tumorosphere formation assays in 3D culture favor the growth and propagation of CSCs from various stages of the disease and allow for screening of different conventional and novel drugs that may focally eradicate these cells (33, 36–39). The importance of these 3D cell culture models is that they enable cell growth in a more physiologically relevant environment than conventional 2D cell cultures (40, 41).

Although the assessment of radiosensitivity in both 2D (the clonogenic assay) and in 3D (sphere formation assay) culture systems can be relevant, very few studies have focused on finding a correlation between them, particularly in the case of bladder cancer (42–45). The purpose of this study is to analyze the response to IR of 3 human bladder cancer cell lines, in 2D and 3D, and to find the correlations between the:

- surviving fraction (SF), or the capacity of irradiated cells to form colonies
- sphere volume (SV), or the capacity of irradiated cells to form spheroids
- and sphere ratio (SR)

while improving our knowledge in the radiobiology of bladder cancer.

## Materials and Methods

### Cell Culture

Three bladder cancer cell lines were used: RT4 (transitional cell papilloma), T24, and UM-UC3 (transitional cell carcinoma). Cells were purchased from the American Tissue Culture Collection (ATCC). Characteristics of each cancer cell line are shown in Supplementary Table 1.

All cells were cultured and maintained in Dulbecco's Modified Eagle Media (DMEM) Ham's F-12 (Sigma-Aldrich) supplemented with 10% heat-inactivated fetal bovine serum (Sigma-Aldrich), 1% Penicillin/Streptomycin (Sigma-Aldrich), and 0.2% plasmocin prophylactic. Cells were incubated at 37°C in a humidified incubator containing 5% CO_2_.

### Irradiation

Cells were irradiated with a 225 kV Precision X-Ray (PXi) irradiator model No X-RAD 225. Irradiation was performed at 3 Gy.min^−1^ and a 1.5 mm Aluminum filter was used.

### Cell Survival Clonogenic (2D) Assay

Cells were plated in 6-well culture plates and incubated at 37°C in a humidified incubator containing 5% CO_2_. After reaching 70% confluency, cells were irradiated at different doses (0 to 10 Gy). Delayed plating technique was performed: 24 h post irradiation, cells were collected and plated in T25 flasks at two different concentrations. The two concentrations were chosen based on the best plating efficiency for each cancer cell line. After incubation for ~10–14 days, cells were fixed with 95% ethanol for 1 min, stained with crystal violet for 3 min, and then washed twice with distilled water. Stained colonies were counted, and each colony was considered as such if formed by more than 50 cells (15, 16, 46, 47).

Surviving fraction (SF) was calculated using the following formula:

(1) $$\begin{array}{r} SF\left(D\right)=\frac{Colonies\;counted}{Cells\;seeded\;\times PE/100} \end{array}$$

SF results were fitted to the Linear-Quadratic model (15–17, 24):

(2) $$\begin{array}{r} \text{SF}\left(\text{D}\right)=\text{exp}\left(-\text{αD}-{\text{βD}}^{2}\right) \end{array}$$

With SF being surviving fraction at a dose D (in Gy) and α(Gy^−1^) and β(Gy^−2^) denoting the adjustment parameters.

### Sphere Formation Assay and Assessing the Ability of Irradiated Cells to Form Spheres (3D Assay)

Although the clonogenic assay is the mainstay of radiosensitivity tests, its main purpose is to assess cells' intrinsic radiosensitivity. Recent advances in cell culture techniques have yielded novel volumetric parameters to characterize cells by growing them in 3D Matrigel™-based cultures and allowing them to form spheres via sphere-formation assay. This commonly used method allows for identifying CSCs and studying their properties *in vitro* (37). Cells' response to radiation can therefore be evaluated by counting the number of spheres as well as the mean volume of spheres after different doses of IR.

Cells were plated in 12-well culture plates at a concentration of 5 × 10^4^ cells per well. After reaching 70% confluency, each plate was treated with a different radiation dose (0 to 10 Gy). Twenty-four hours post-irradiation, cells were trypsinized, collected, counted, and seeded as single cells in Matrigel™/DMEM Ham's F-12 (serum-free) (1:1) at a concentration of 2 × 10^3^ cells/well in a total volume of 50μl in duplicates, as previously described (34, 36–38). Cells were then plated around the rim of the wells of 24-well culture plates and allowed to solidify in the incubator for 45 min. DMEM Ham's F-12 media with 3% heat-inactivated fetal bovine serum was then added to the center of the wells.

Depending on the cell line, the spheres were counted on a Zeiss Axio Vert.A1 microscope, and their sizes were measured 5–7 days post irradiation with the Zen 2.3 lite blue edition software. Sphere numbers and diameter sizes were assessed for each sphere with a minimum diameter of 40μm, as previously described by Sart et al. (48). Sphere ratio (SR) was calculated using the following equation:

(3) $$\begin{array}{r} SR\left(D\right)=\frac{Number\;of\;spheres\;at\;dose\;D}{Number\;of\;spheres\;at\;0Gy} \end{array}$$

### Sphere Forming Unit (SFU) Definition

SFU was calculated based on this formula (37):

(4) $$\begin{array}{r} SFU\left(\%\right)=\frac{number\;of\;spheres\;counted}{number\;of\;spheres\;seeded}\times 100 \end{array}$$

For example, the formation of 80 spheres after plating 2,000 cells indicates an SFU of 4%.

### Sphere Formation Inhibition

Sphere formation inhibition was calculated using the below equation:

(5) $$\begin{array}{r} SI\left(D\right)=\frac{SFU\left(0Gy\right)-SFU\left(D\right)}{SFU\left(0Gy\right)}\times 100 \end{array}$$

SI, as a function of dose, was fitted to the below equation:

(6) $$\begin{array}{r} SI\left(D\right)=100\times \left(1-\exp\left(-aD\right)\right) \end{array}$$

With SI being the percentage of sphere inhibition at a dose D in Gy, and a (Gy^−1^) denoting a fitting parameter that describes the increase in inhibition with the dose.

### Sphere Volume Assessment

Volume was then calculated using the below equation:

(7) $$\begin{array}{r} V=\frac{4\pi }{3}\times {\left(\frac{d}{2}\right)}^{3} \end{array}$$

With V being the volume and d the diameter

Volume as a function of the dose was fitted to a negative exponential model:

(8) $$\begin{array}{r} V\left(D\right)=V\left(0\right)\exp\left(-bD\right) \end{array}$$

With V being the volume in μm^3^ at a dose D (in Gy), and b (Gy^−1^) denoting a fitting parameter that describes the decrease rate with the dose.

VR was calculated using the following equation:

(9) $$\begin{array}{r} VR\left(D\right)=\frac{V_{0}-V\left(D\right)}{V_{0}}\times 100 \end{array}$$

With V_0_ being the volume without exposure to IR, V(D) the volume after a treatment with a dose D in Gy.

VR, as a function of dose, was fitted to a curvilinear model:

(10) $$\begin{array}{r} VR\left(D\right)=VR_{\max}\left(1-\exp\left(-eD\right)\right) \end{array}$$

With VR being the percentage of volume reduction, VRmax being the maximal volume reduction, and e (Gy^−1^) being a fitting parameter.

### Statistical Analysis

Data and statistical analysis was done using MATLAB R2016a (MathWorks, Natick, MA). ANOVA test was performed to validate the differences between volumes after irradiation.

A linear regression was performed to assess the potential correlation between 2D and sphere results. A multiple regression model correlating survival fraction (SF), sphere ratio (SR) and volume reduction (VR) was modeled with the following function:

(11) $$\begin{array}{r} SF\left(D\right)=\;X_{0}+X_{1}\times SR\left(D\right)+X_{2}\times VR\left(D\right) \end{array}$$

The data fit for SF(D), SR(D), V(D), and VR(D) by Equations (2), (6), (8), and (10) was obtained by minimizing the least squares residual. The algorithm used was the trust-region-reflective optimization which is based on the interior-reflective Newton method (49). The least squares calculations were obtained by using the lsqcurvefit command in Matlab Software (The Mathwork, Natick, MA, USA), and were stopped when the final change in the sum of squares relative to its initial value became less than the default value of the function tolerance.

*R*^2^ values, also known as the coefficient of determination, a statistical measure of how close the data are to the fitted regression line, were calculated automatically with the cited algorithm for all fits: values range from 0 to 1, with 1 being the perfect fit.

## Results

### Cell Survival Results: Clonogenic (2D) and Spheres-Formation (3D) Assays

In order to assess the radio-induced cell death of the three human bladder cancer cell lines, clonogenic assay was employed.

Our results were in consonance with this model (**Figure 3**) with *R*^2^ values of 0.99, 0.99, and 0.98 for RT4, T24, and UM-UC-3, respectively. It is also noteworthy mentioning that RT4 was the most resistant bladder cancer cell line with a SF of 0.54 ± 0.07 at 2 Gy (SF2), whereas T24 and UM-UC-3 cells had SF2 values of 0.38 ± 0.09 and 0.35 ± 0.07, respectively. Fitting parameters and coefficients are shown in Supplementary Table 2. To ensure the phosphorylation of H2AX histone variants (γ-H2AX) following IR, immunofluorescence was used. Representative examples of nuclei stained by γ-H2AX antibodies and counterstained by DAPI at 2 Gy (10 min) vs. 0 Gy for RT4, T24, and UM-UC-3 cells are shown in Supplementary Figure 1.

**Figure 1:**
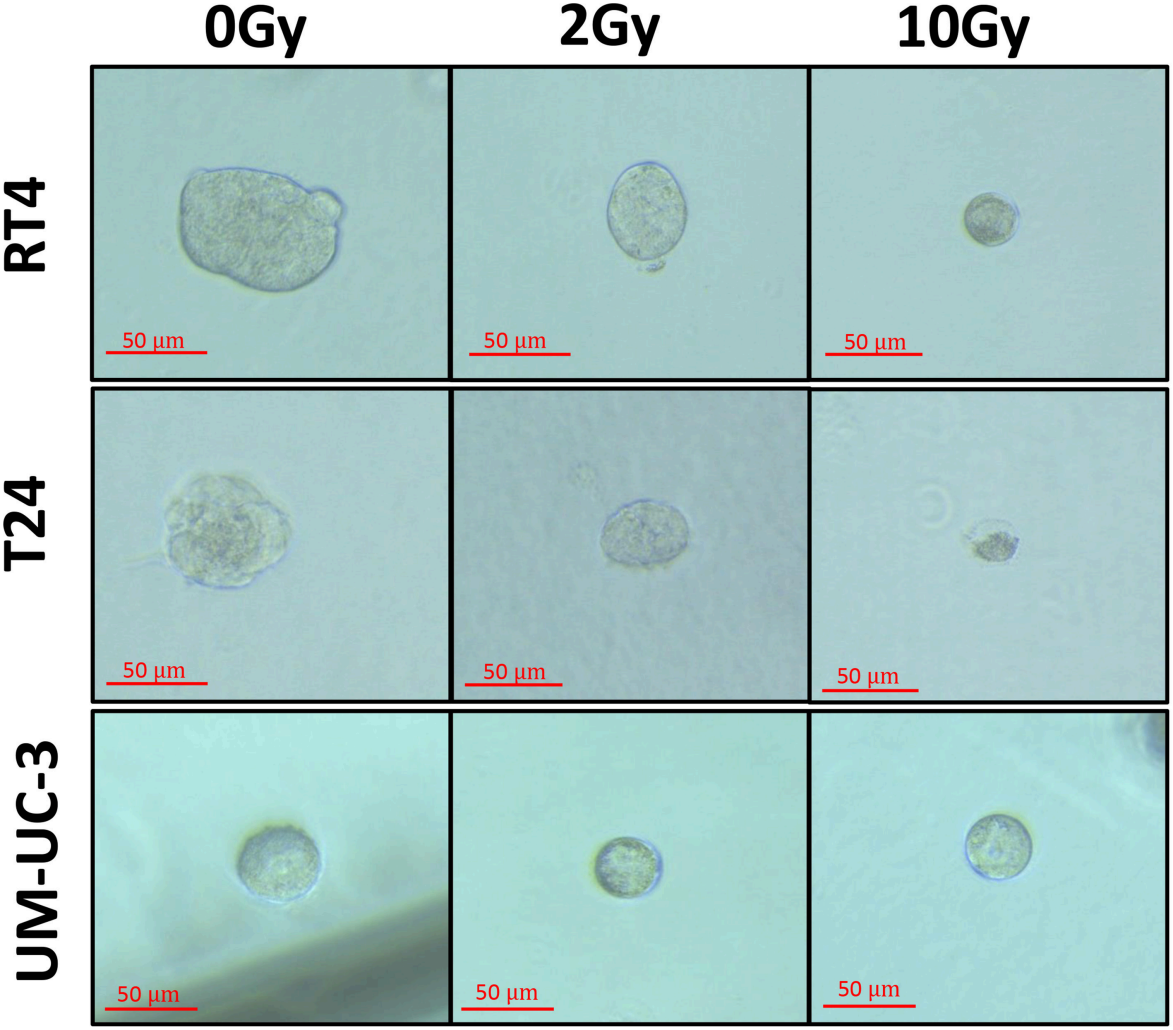
Morphology of human bladder cancer cells in 3D. Representative bright-field images of RT4, T24, and UM-UC-3 spheres with or without radiation treatment (at 0, 2, and 10 Gy). Images were visualized by Zeiss Axiovert inverted microscope at 10x magnification and analyzed by Carl Zeiss Zen 2012 image software. Scale = 50 μm.

**Figure 2:**
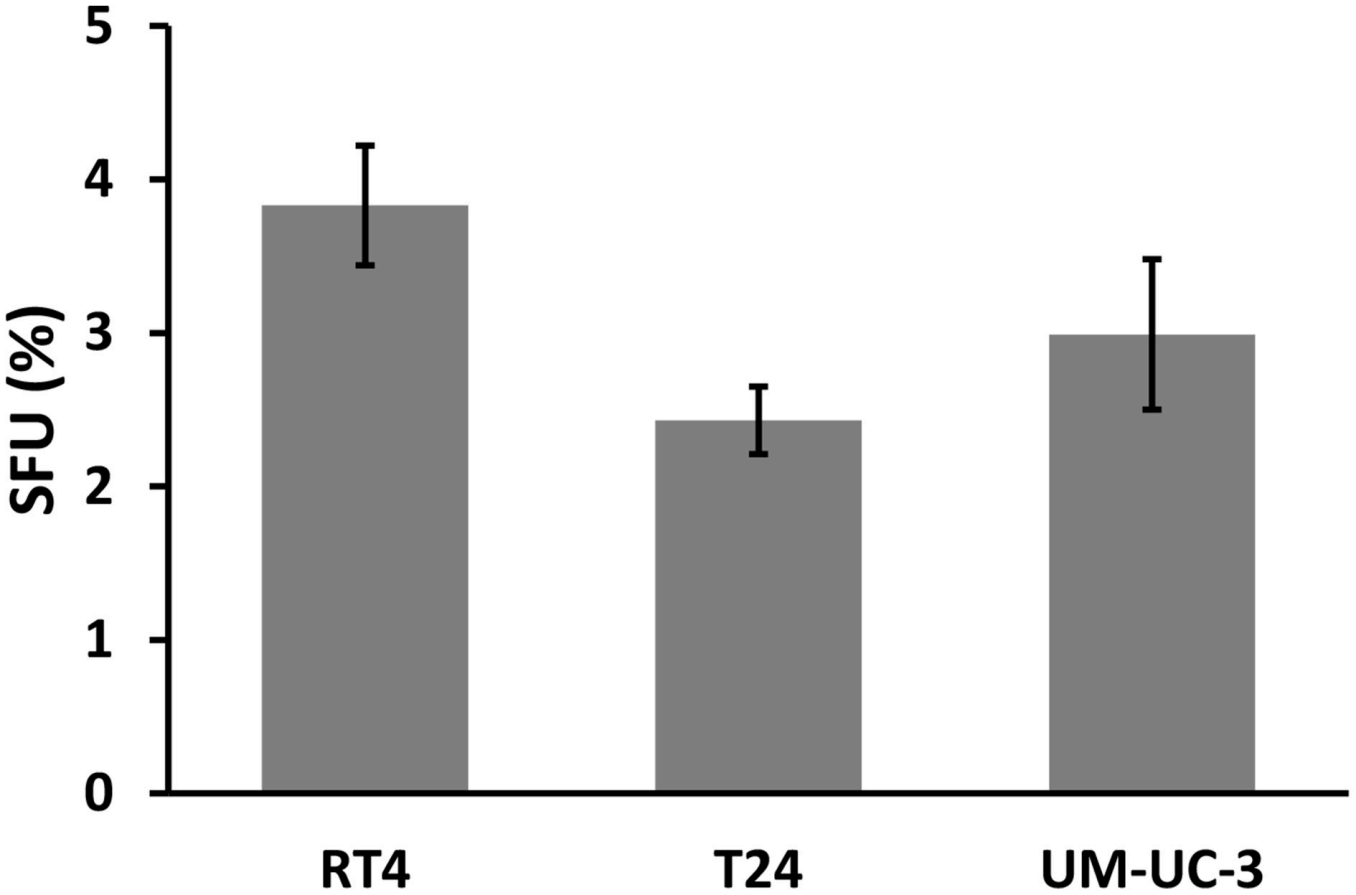
SFU of the 3 human bladder cancer cell lines without IR. Results represent the mean of at least three independent experiments of sphere-formation assay ± standard error of the mean (SEM).

**Figure 3:**
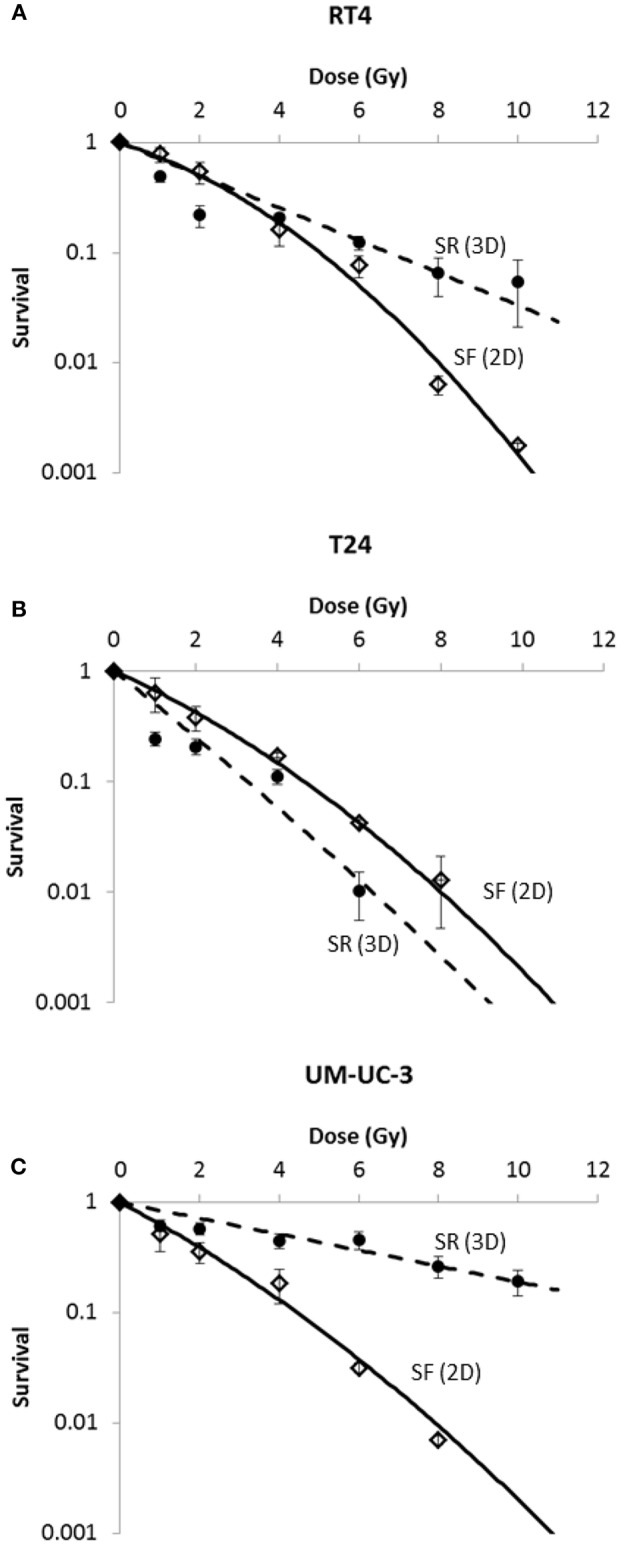
Radia-induced cell death in 2D (SF) and 3D (SR) for the 3 human bladder cancer cell lines. SR was calculated using the equation $R\left(D\right)=\frac{SFU\left(D\right)}{SFU\left(0Gy\right)}$. Each data point represents the mean value of at least three independent experiments ±SEM. Data are fitted to a linear quadratic (LQ) model. Fitting parameters and the corresponding R^2^ values are shown in Supplementary Tables 2,3.

**Figure 4:**
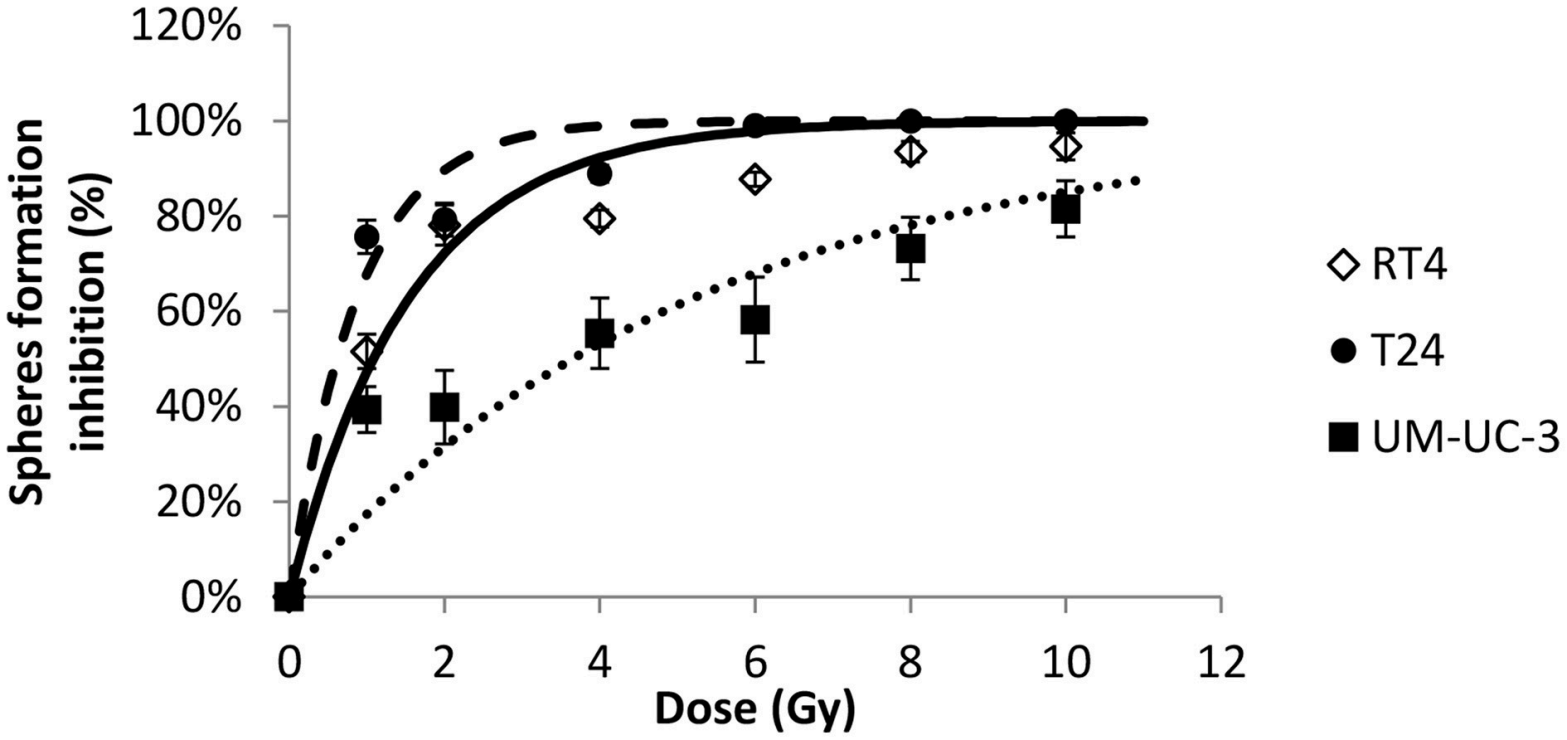
Radiation-induced decrease of number of spheres for the 3 human bladder cancer cell lines. Each data point represents the mean value of at least three independent experiments ±SEM. Sphere formation inhibition was calculated using the equation $SI\left(D\right)=\frac{SFU\left(0Gy\right)-SFU\left(D\right)}{SFU\left(0Gy\right)}\times 100$. Data are fitted to a curvilinear model *SI*(*D*) = 100 × (1 − exp(−*aD*)). *R*^2^ values for RT4, T24, and UM-UC-3 are respectively, 0.94, 0.96, and 0.84. Fitting parameters and the corresponding *R*^2^ values are shown in Supplementary Table 4.

Next, we sought to determine the relationship between the number of spheres and the dose. Without IR, SFU was 3.83 ± 0.4%, 2.43 ± 0.22%, and 3 ± 0.5% for RT4, T24, and UM-UC-3, respectively (Figures 1 and 2). After IR, a clear difference was observed in reduction of the SR (Figure 3). The outcomes of 3D cultures displayed a proper fit with the Linear-Quadratic model, whereby the equation used for cell survival in 2D was found to be relevant for the 3D results as well, with *R*^2^ values of 0.85, 0.9, and 0.84 for RT4, T24 and UM-UC-3 cells, respectively. Nonetheless, the radiosensitivity status evinced here contradicts that of the clonogenic assay results, where UM-UC-3 exhibited the highest radioresistance in its capacity to form spheres with 57 ± 7% remaining spheres in response to 2 Gy (Figure 3C), while this value was 22 ± 5% and 21 ± 3.5% for RT4 and T24, respectively (Figures 3A,B). This effect can be also observed when plotting sphere inhibition with the dose [Equation (6) and Figure 4], where RT4 and T24 spheres formation was completely inhibited after a 10 Gy dose, while only 82 ± 6% of the UM-UC-3 spheres was inhibited at this high dose. Besides, even when the SF reached the 0 value, UM-UC-3 cells were able to maintain 30% of the spheres at 8 Gy and 18% at 10 Gy.

In addition, results showed that the difference in radiosensitivity between the 2D and 3D culture systems is highly dependent on the cell line:

- For RT4, cells were more sensitive in 3D at lower doses but became more resistant at higher doses (Figure 3A).
- For T24, cells were more sensitive when cultured in 3D (Figure 3B).
- For UM-UC-3, cells were more resistant in 3D (Figure 3C).

**Figure 5:**
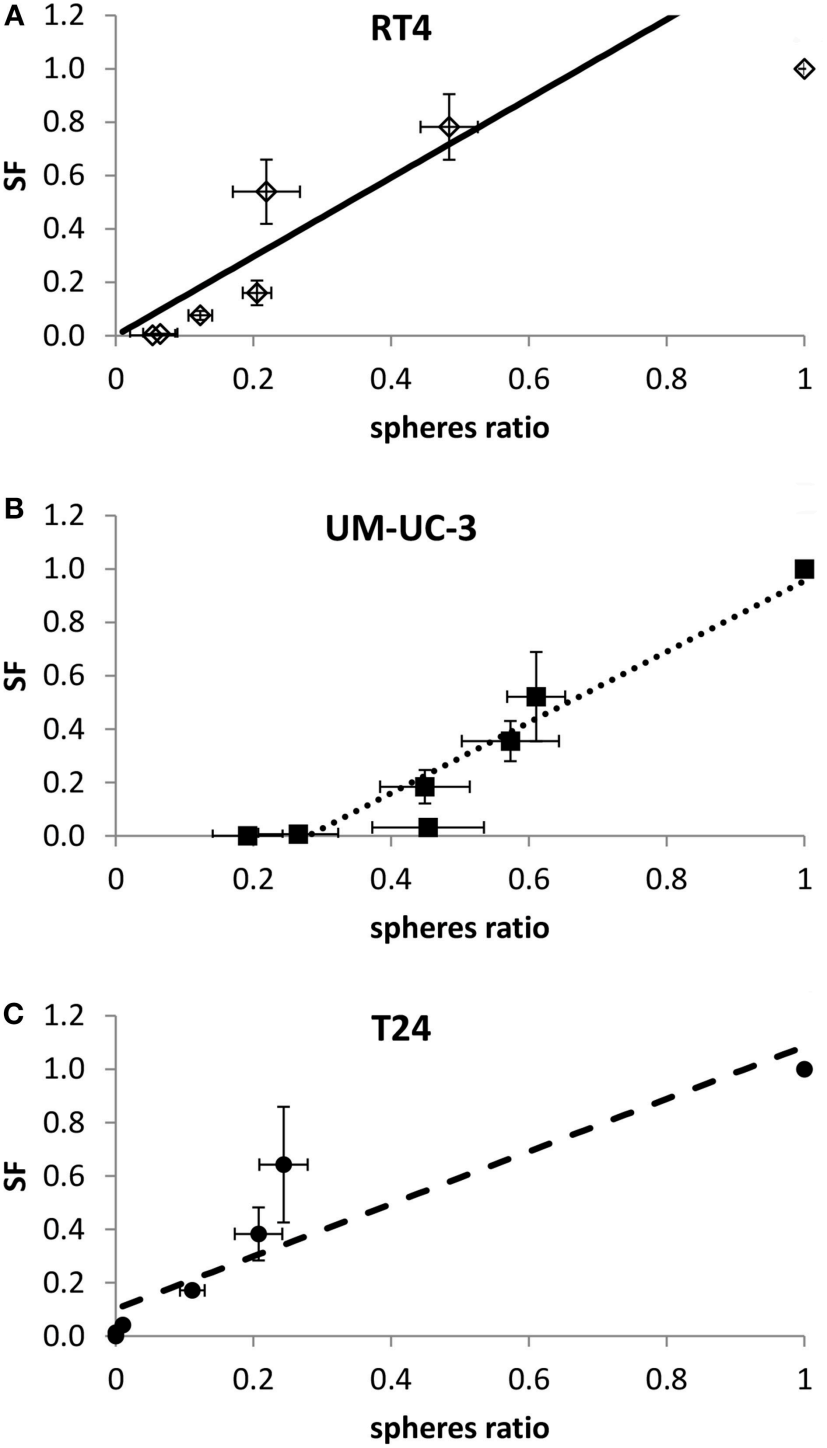
Linear correlation between SF and sphere ratio for each cell line. Each data point represents the mean of at least three independent experiments ±SEM. (**A)** RT4 cell line (*R*^2^ = 0.84). (**B)** T24 cell line (*R*^2^ = 0.85). (**C)** UM-UC-3 cell line (*R*^2^ = 0.92). Data are fitted to a linear model. Fitting parameters and the corresponding *R*^2^ values are shown in Supplementary Table 5.

After showing the evolution of SF and SR with the dose, a linear regression was performed to assess the potential correlation between 2D and sphere results. A linear correlation was found between SR and SF for the 3 cancer cell lines, with *R*^2^ coefficients of 0.84, 0.85, and 0.92 for RT4, T24, and UM-UC-3, respectively (Figure 5).

### The Effect of Sphere Volume Without Irradiation Is Dominant When Assaying Volume Reduction (VR)

**Figure 6:**
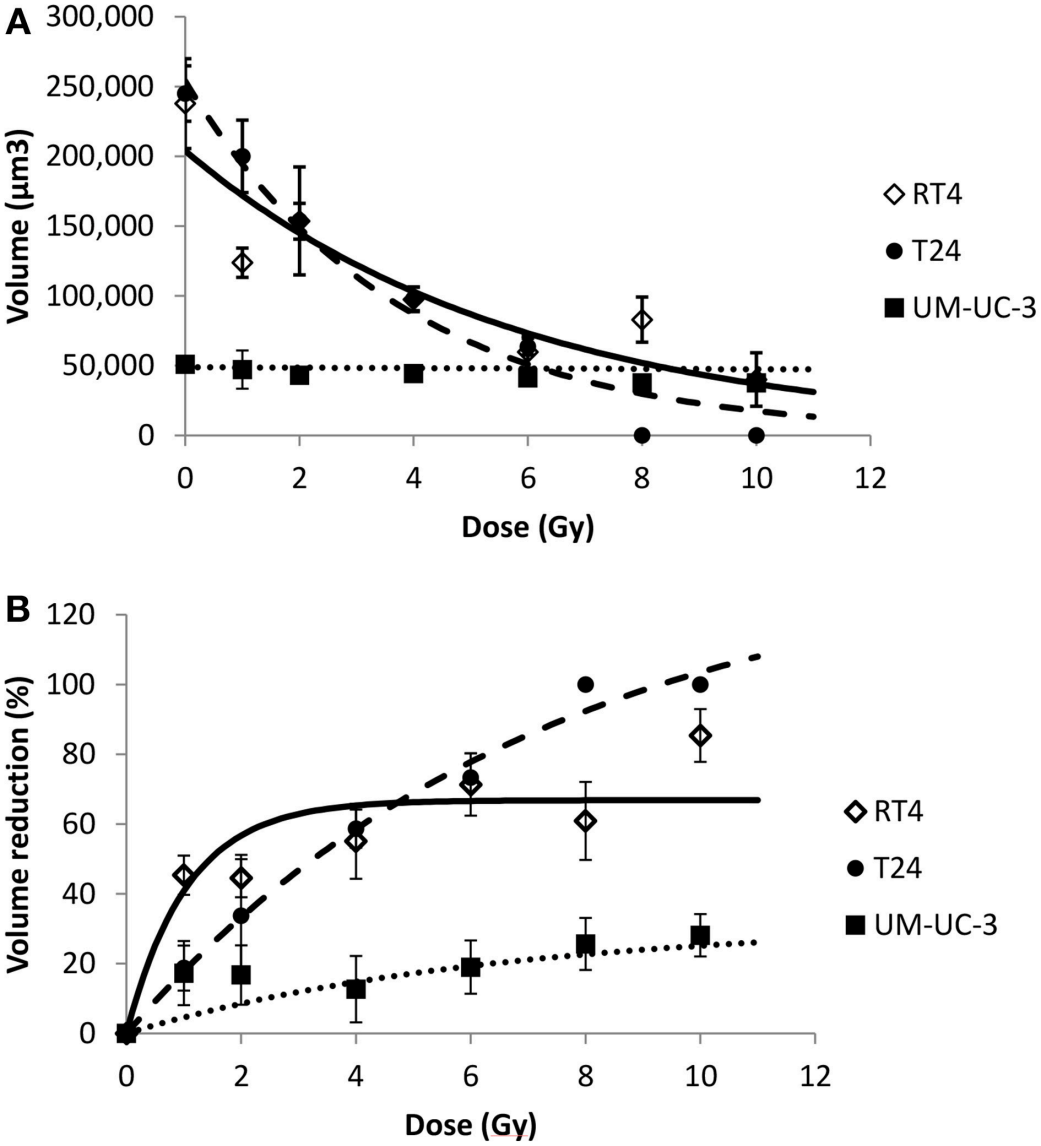
Radiation-induced sphere volume decrease for the 3 human bladder cancer cell lines. Each data point represents the mean value of at least three independent experiments ±SEM. (**A)** spheres volume. Data are fitted to a negative exponential model: *y* = *V*(0)exp(−*bx*). *R*^2^ values for RT4, T24, and UM-UC-3 are respectively, 0.82, 0.97, and 0.88. Fitting parameters and the corresponding *R*^2^ values are shown in Supplementary Table 6. **(B)** sphere volume reduction ($\frac{V\left(0Gy\right)-V\left(D\right)}{V\;\left(0Gy\right)}\times 100$). Data are fitted to a curvilinear model: *y* = *VR*_max_ × (1 − exp(−*ax*)). *R*^2^ values for RT4, T24, and UM-UC-3 are respectively, 0.9, 0.99, and 0.81. Fitting parameters and the corresponding *R*^2^ values are shown in Supplementary Table 7.

Since sphere formation assay has been widely used in different cancers for the isolation and enrichment of CSCs, phenotypes of those CSCs differ among the various cell lines used yielding a heterogeneity in the average number, i.e., SFU, as well as the mean size of spheres formed (50). In our study, without irradiation, RT4 and T24 cancer cell lines were able to form spheres of comparable volume sizes, 24 ± 3.2 × 10^4^ and 24 ± 2 × 10^4^ μm^3^, respectively, while much smaller spheres were formed in UM-UC-3 cells, with an average volume of 5 ± 0.58 × 10^4^ μm^3^ (Figures 1 and 6A).

For the 3 cancer cell lines, volume followed a negative exponential model [Equation (8)].

*R*^2^ values for RT4, T24, and UM-UC-3 cells were as follow: 0.82, 0.97 and 0.88, respectively (Figure 6A).

On the other hand, VR(D) followed a curvilinear shape [Equation (10)] for the three cell lines with *R*^2^ coefficients for RT4, T24, and UM-UC-3 of 0.9, 0.99, and 0.81, respectively (Figure 6B).

The effect of the initial sphere size on VR with the dose was very dominant, with UM-UC-3 having the least efficient VR, while the volume decreased significantly for both RT4 and T24 (Figures 6A,B). This is probably due to the fact that the diameter limit for a spheroid to be considered as such was 40 μm, as previously described by Sart et al. (48), which corresponds to a volume of 3.3 × 10^4^ μm^3^. This means that any UM-UC-3 sphere with a reduction of more than 30% will not be taken into account.

To confirm this observation, we pooled the sphere volume data for each dose and each cancer cell line to be able to perform an ANOVA analysis and verify the volume decrease with the dose. A significant sphere VR was observed for RT4 and T24 cancer cell lines at all the doses and the control (*P* < 0.01). For UM-UC-3 cells, however, no significant decrease was observed between the different irradiation doses (*P* > 0.05), and the only acceptable difference was between the control (0 Gy) and the irradiated cells (*P* = 0.03).

It is also noteworthy mentioning that the reduction percentage was higher for RT4 at lower doses ranging between 1 and 4 Gy. On the other hand, VR was more pronounced at higher doses for T24 cells, reaching a value of 100% at 8 and 10 Gy (Figures 6A,B).

### Correlation Between Sphere Ratio (SR) and Sphere Volume

**Figure 7:**
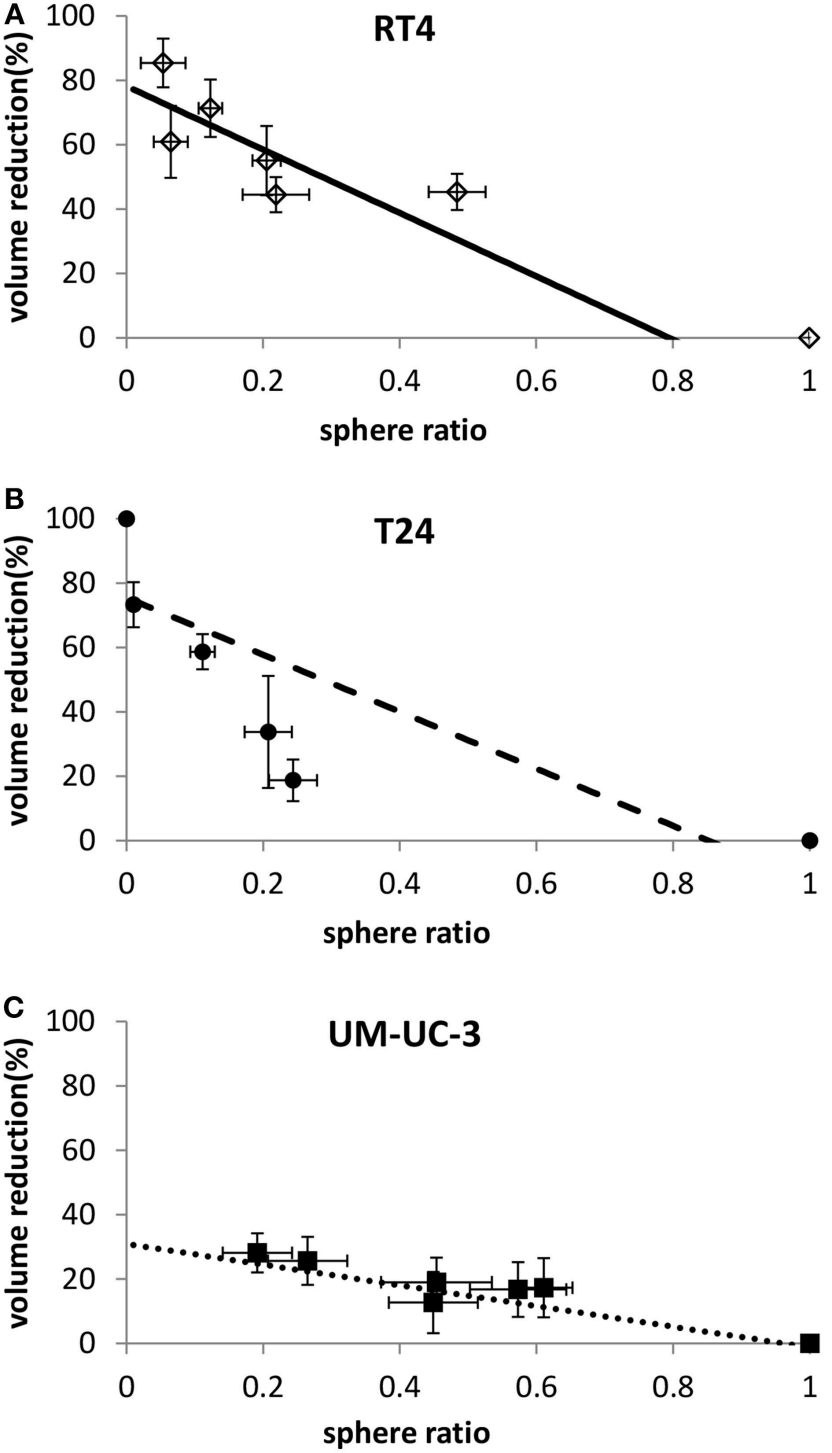
Linear correlation between sphere volume reduction and sphere ratio for each cell line. Each data point represents the mean value of at least three independent experiments ±SEM. (**A)** RT4 cell line (*R*^2^ = 0.9). (**B)** T24 cell line (*R*^2^ = 0.64). (**C)** UM-UC-3 cell line (*R*^2^ = 0.9). Data are fitted to a linear model. Fitting parameters and the corresponding *R*^2^ values are shown in Supplementary Table 8.

To check whether sphere VR and SR values are independent or proportional, both variables were plotted against each other (Figure 7). RT4 and UM-UC-3 showed a linear correlation between volume and number with *R*^2^ = 0.9 for both cancer cell lines. Although the volume data for UM-UC-3 were not significant as mentioned previously, at least for RT4, sphere volume and sphere number are responding in the same way to IR (Figures 7A,C). On the other hand, correlation coefficient of T24 (R^2^ = 0.64) showed no linear correlation between those 2 parameters (Figure 7B).

### Combining Sphere Ratio (SR) and Volume Regression (VR) to Predict Radiosensitivity

Lastly, we evaluated the possibility of correlating a function combining both 3D parameters, SR and VR, with the SF in 2D, and how this function can improve the quality of the correlation. As previously mentioned, SR and VR are proportional for RT4 and UM-UC-3, so combining both can add little to no value. However, it was interesting to perform a linear regression for T24, knowing that the radiosensitivity information that was obtained from the volume differs from that obtained from the SR. Accordingly, we developed a multiple regression model with Equation (9).

*R*^2^ coefficient was improved and had a value of 0.97 for the combined parameters, while it was 0.85 when taking the SR alone and 0.89 when taking the VR alone. In addition to that, both parameters demonstrated a significant impact as the *p*-values were lower than 0.05 (Supplementary Table 9).

The same model was applied for RT4 and UM-UC-3 but no significant improvement was observed and only one out of the 2 parameters had a significant impact (*P* < 0.01, data not shown).

## Discussion

Individual radiosensitivity of tumors and normal tissues have always been a big challenge for clinicians and radiobiologists (51). For normal tissues, radiosensitivity is defined by radio-induced toxicities, while for tumors the main parameter is tumor control. Many predictive tools are still being developed today with the main purpose of trying to understand a patient's response to the treatment (13, 23, 52–59). Those involve functional assays that are mainly based on DNA repair (56–58), apoptosis (60), proteomics (61), and radiogenomics (62).

On the other hand, cellular radiosensitivity is defined by the capacity of irradiated cells to produce colonies, which is measured by performing the clonogenic assay (15). The LQ model is considered as the best-fitting model to describe survival and of great interest in radiation oncology through the link existing between the α/β ratio and the nature of radiotherapy-induced tissue (early or late) reactions and tumor response to fractionation (63–67). Recently, a new interpretation of the LQ model was proposed based on the nucleo-shuttling of the ATM protein (17, 24).

By considering that the clonogenic assay shows only the intrinsic radiosensitivity of a given cell line, one can argue that the radio-response might be modified by adding other factors and constraints. Indeed, cells grown in 2D monolayers might respond in a different manner if they were grown in 3D, and for different reasons like hypoxia, cell density, and most of all stem cells radiosensitivity. It was therefore interesting to understand the relationship between the response to IR of cells grown in 2D and 3D.

With the emergence of new bladder preserving cancer treatment strategies relying on radiotherapy, a radiobiological characterization of bladder cancer is becoming a necessity (4, 68–70). Many studies focused on the effects of IR on bladder cell survival in 2D or on bladder chemosensitivity in 3D cell culture, but to date there is no biological endpoint to predict bladder cancers' response to IR (2, 11, 71, 72). In this study we showed that implementing 3D cell cultures can be a useful tool to assess tumor radiosensitivity by taking into account the volumetric constraints.

When cultured in monolayers, RT4 cancer cell line (transitional cell papilloma) was shown to be the most radioresistant, while the two transitional cell carcinoma cell lines were more sensitive and had a comparable response to radiation. Results in 3D were different, as the UM-UC-3 cancer cell line was the most resistant in both VR and SR, followed by RT4 and T24. While VR for UM-UC-3 was not statistically significant, it was interesting to see that, even after 8 or 10 Gy, more than 20% of the spheres survived. The modification of radiosensitivity status can also be seen in Figure 4C: even when SF was equal to zero, we still have surviving spheres for this specific cell line. This can be explained by the effect of multiple additional factors. The effect of Matrigel™ in facilitating cell growth has been observed in many studies and might help the cells proliferate after IR (73, 74). The relative effect of Matrigel™ might vary from one cell line to the other. Another factor that must be taken into account is that in 3D cell culture, the specific population of stem cells is targeted, which is not necessarily the case in the clonogenic assay (42, 75). Radiosensitivity of bladder stem cells can be different than that of other cells (42, 68, 75, 76).

Another interesting observation is that in the case of both RT4 and UM-UC-3, spheres were more resistant than cells in monolayers for doses higher than 2 Gy, while it was the other way around for T24. This can also be explained by the difference in radiosensitivity of CSCs, as they were shown to be more radioresistant than the average cell in RT4 and UM-UC3, and more sensitive in T24. This finding can help us in finding the best compromise for tumor control and cancer recurrence by combining 2D and 3D results: indeed, as it was mentioned previously, SF is correlated with tumor control dose, while the radiosensitivity of CSCs is generally correlated with recurrence and treatment failures (77, 78). For instance, a fraction of 4 Gy shows close survival in 2D and 3D for RT4 and T24 cancer cell lines (0.1 ± 0.02 vs. 0.16 ± 0.04 and 0.11 ± 0.02 vs. 0.17 ± 0.01, respectively), while spheroids were more sensitive for both cell lines at lower doses. For higher doses however, RT4 spheres became more resistant, leading therefore to a higher possibility of recurrence when compared with tumor control. The case of UM-UC-3 was different as it spheres were more resistant at all doses.

Another interesting endpoint for 3D spheroids is the sphere volume after IR. The volume might highlight the capacity of the stem cell to regenerate, and from a clinical perspective, it might be translated into the speed or severity of the recurrence. Our results are showing that RT4 is the most radiosensitive when it comes to VR, followed by T24 and finally UM-UC-3.

Lastly, we asked whether radiosensitivity in 3D can be described by sphere volume or SFU. Cells cultured in 3D have a more complex micro-environment than when cultured in monolayers. In addition to the ratio of surviving spheres, VR gives us additional information about the cell's response to IR. Interestingly, our results are showing that radiosensitivity can be described in terms of VR and sphere formation in different manners that are also intrinsic to the cell line:

- For RT4: Either SR or VR can be used to describe the radio-response.
- For T24: Both parameters are required to explain the response to radiation (Figure 7B).
- For UM-UC-3: Only SR must be considered to describe radiosensitivity (Figure 7C).

Indeed, even though 3D culture is promising, it still requires more extensive research. In particular, a 3D model that better represents the *in vivo* environment must be developed in order to take in consideration cell-cell signaling, cell-ECM interaction, the heterogeneous nature of tissues and the vascular network that supplies a tissue (79).

Collectively, our findings indicate that in addition to the differences in intrinsic radiosensitivity, the outcome of the different parameters assessed in 3D also varies with the cell line, and therefore we propose that both 2D and 3D model assays should be performed to better assess and predict response to ionizing radiation. Those results presented here depict a cornerstone for future assays looking at radiosensitivity of primary cultures from bladder cancer patients. Such tests might help clinicians in choosing the most adapted treatment for each patient: patients with radiosensitive tumors can be candidates to bladder cancer preserving strategies involving radiotherapy, while those with more radioresistant cancers will have to undergo cystectomy.

To conclude, bladder cancer patients are usually treated with multi-fractionated radiotherapy (i.e., dose fractionation), with fractions ranging between 1.8 and 2.5 Gy and total treatment doses reaching 70 Gy in some cases (80); this underscores the need for future studies to assess radiosensitivity of bladder cancer cells while taking into consideration dose-fractionation as a mode of treatment. Besides, evaluating bladder cancer radiosensitivity using *in vivo* could also be performed to validate our results in pre-clinical animal models.

## Study Limitations

Limitations in our study reside in the diameter limit for a spheroid to be considered as such, where spheres <40 μm diameter (corresponding to a volume of 3.3 × 10^4^ μm^3^) were not included in the analysis (48). Therefore, UM-UC-3 spheres that had a volume reduction of more than 30% were not taken into account. However, this limit was applied for all the doses and all the cell lines. Although one would argue that if we lowered the limit to 30 μM for example, the number of spheres would grow, and the mean volume would decrease. Yet, this will be proportional for all doses, leading to the same SR and VR after normalization.

## Author Contributions

LB, HB, TA, JA, JB-G, and KC contributed to the project design and execution of experiments, analysis of results, and writing of manuscript. LB, YZ, TE, FG, and WA-K contributed to overlooking and following up with experiments, result analysis, and manuscript proof reading. LB and WA-K contributed to project design, result analysis, manuscript writing, and proofreading.

### Conflict of Interest Statement

The authors declare that the research was conducted in the absence of any commercial or financial relationships that could be construed as a potential conflict of interest.

## Supplementary Material

Supplementary Figure 1 Representative examples of nuclei stained by γ-H2AX antibodies and 814 counterstained by DAPI at indicated conditions for RT4, T24, and UM-UC-3. Images were taken 815 with Zeiss LSM 710 Laser confocal microscope at 100×.

Supplementary Table 1 Cell lines specifications and cancer-associated gene alterations pertaining to each cell line.

Supplementary Table 2 Clonogenic assay results and fitting. LQ model was used with SF(D) = exp(−αD−βD^2^). Coefficients are shown with with 95% confidence bounds.

Supplementary Table 3 SR results and fitting parameters. LQ model was used with SF(D) = exp(−αD−βD^2^). Coefficients are shown with with 95% confidence bounds.

Supplementary Table 4 Sphere inhibition results and fitting parameters. The model used was SI(D)=1 − exp(−aD). Coefficients are shown with with 95% confidence bounds.

Supplementary Table 5 SR vs SF linear correlation parameters. The model used was *SF*(*D*) = *f* × *SR*(*D*) + *g*. Coefficients are shown with with 95% confidence bounds.

Supplementary Table 6 Volume results and fitting parameters. The model used was *V*(*D*) = *V*(0)*exp*(−*bD*). Coefficients are shown with with 95% confidence bounds.

Supplementary Table 7 VR results and fitting parameters. The model used was *VR*(*D*) = *VR*_max_(1 − exp(−*eD*)). Coefficients are shown with with 95% confidence bounds.

Supplementary Table 8 VR vs SR linear correlation parameters. The model used was *R*(*D*) = *h* × *SR*(*D*) + *k*. Coefficients are shown with with 95% confidence bounds.

Supplementary Table 9 Multiple regression model correlating SF, SR and VR for T24 cell line. The model used was: *SF*(*D*) = *X*_0_ + *X*_1_ × *SR*(*D*) + *X*_2_ × *VR*(*D*).

## Abbreviations

ATCC: American Tissue Culture Collection
CSC: cancer stem cells
DMEM: Dulbecco's Modified Eagle Media
IR: ionizing radiation
SF: surviving fraction
SFU: sphere forming unit
SR: sphere ratio
VR: volume reduction/regression
2D: two-dimension
3D: three-dimension.
