# Supplementary figures and images for: Assessing Radiosensitivity of Bladder Cancer in vitro: A 2D vs. 3D Approach
Source: Front Oncol. 2019 Mar 19;9:153. doi: 10.3389/fonc.2019.00153 (PMC6433750; doi:10.3389/fonc.2019.00153)

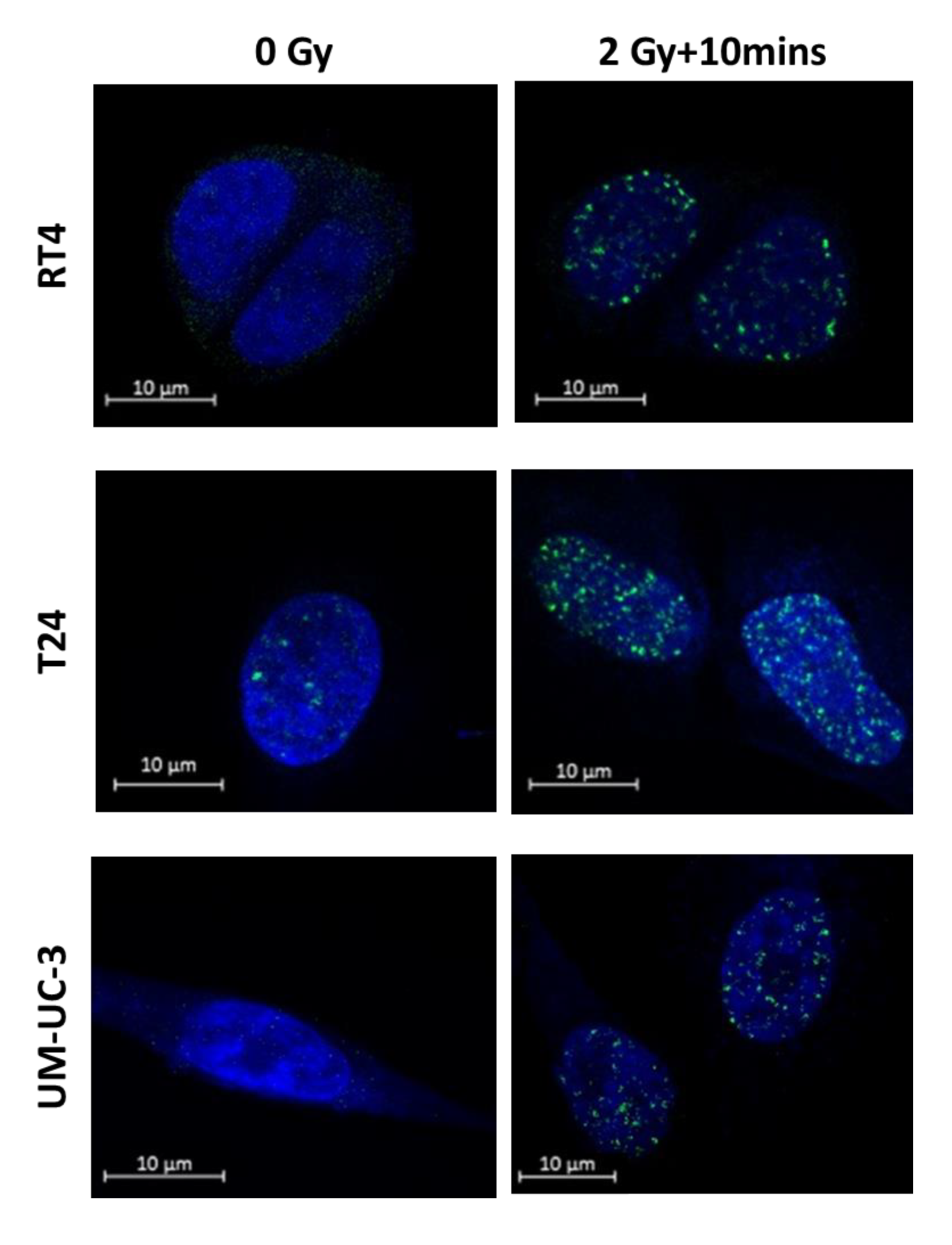

Supplement: Supplementary Figure 1 — Representative examples of nuclei stained by γ-H2AX antibodies and 814 counterstained by DAPI at indicated conditions for RT4, T24, and UM-UC-3. Images were taken 815 with Zeiss LSM 710 Laser confocal microscope at 100×. [file Image_1.TIF]
