# Supplementary material for: Assessing Radiosensitivity of Bladder Cancer in vitro: A 2D vs. 3D Approach
Source: Front Oncol. 2019 Mar 19;9:153. doi: 10.3389/fonc.2019.00153 (PMC6433750; doi:10.3389/fonc.2019.00153)
Supplement: Supplementary Table 1 — Cell lines specifications and cancer-associated gene alterations pertaining to each cell line. [file Data_Sheet_1.PDF]

Supplementary Tables

Table 1: Cell lines specifications and cancer-associated gene alterations pertaining to each cell line.

Cell Line	Tissue	Disease	Age	Cancer-Associated Gene Alterations (Nickerson et al., 2017)			
				TP53	***TERT***	***MLL3/ KMT2C***	***TSC1***
**RT4**	Urinary bladder	Transitional cell papilloma	63 years	-	UTR, promoter, and splice site alterations	-	Coding indels
**UM-UC-3**	Urinary bladder	Transitional cell carcinoma	-	Missense	UTR, promoter, and splice site alterations	-	-
**T24**	Urinary bladder	Transitional cell carcinoma	81 years	Nonsense	UTR, promoter, and splice site alterations	Missense	-

Abbreviations: *TP53: tumor protein p53; MLL3/KMT2C: Histone-Lysine N-Methyltransferase 2C; TERT: telomerase reverse transcriptase; TSC1: tuberous sclerosis 1.*

Table 2: clonogenic assay results and fitting. LQ model was used with $\mathrm{SF(D)} = \exp(-\alpha \mathrm{D} - \beta \mathrm{D}^2)$. Coefficients are shown with 95% confidence bounds.

Bladder cell lines	SF2	α(Gy^{-1})	β(Gy^{-2})	R^2
**RT4**	0.54 (±0.07)	0.26 (0.057, 0.47)	0.039 (0.014, 0.063)	0.99
**UM-UC-3**	0.36 (±0.07)	0.26 (0.068, 0.46)	0.05 (0.027, 0.073)	0.98
**T24**	0.38 (±0.09)	0.4 (0.31, 0.50)	0.018 (0.0035, 0.032)	0.99

Table 3: SR results and fitting parameters. LQ model was used with $\mathrm{SF(D)} = \exp(-\alpha \mathrm{D} - \beta \mathrm{D}^2)$. Coefficients are shown with 95% confidence bounds.

Cell line	α(Gy^{-1})	β(Gy^{-2})	R^2
**RT4**	0.034 (0.27, 0.41)	0	0.85
**T24**	0.68 (-0.28, 1.6)	0.0082 (-0.18, 0.19)	0.9
**UMUC3**	0.17(0.13, 0.2)	0	0.88

Table 4: Sphere inhibition results and fitting parameters. The model used was SI(D)=1-exp(-aD). Coefficients are shown with 95% confidence bounds.

Cell line	a(Gy^{-1})	R^2
**RT4**	0.64 (0.4, 0.87)	0.94
**T24**	1.1 (0.69, 0.4)	0.96
**UMUC3**	0.19 (0.1228, 0.2571)	0.84

Table 5: SR vs SF linear correlation parameters. The model used was $SF(D) = f \times SR(D) + g$. Coefficients are shown with 95% confidence bounds.

Cell line	f	g	R^2
**RT4**	1.5 (1, 1.9)	0	0.84
**T24**	1 (0.51, 1.4)	0.1 (-0.09, 0.3)	0.85
**UMUC3**	1.3 (0.88, 1.8)	-0.37 (-0.52, -0.11)	0.92

Table 6: Volume results and fitting parameters. The model used was $V(D) = V(0)exp(-bD)$. Coefficients are shown with 95% confidence bounds.

Cell line	V(0)x10^4 (μm^3)	b (Gy-1)	R^2
**RT4**	6.5 (4.6, 8.4)	0.18 (0.28, 0.06)	0.82
**T24**	8.1 (6.8, 9.3)	0.27 (0.35, 0.18)	0.97
**UMUC3**	1.5 (1.4, 1.6)	0.029 (0.017, 0.041)	0.88

Table 7: VR results and fitting parameters. The model used was

$VR(D) = VR_{max}(1 - exp(-eD))$. Coefficients are shown with 95% confidence bounds.

cell line	VR_{max} (%)	e (Gy^{-1})	R^2
**RT4**	66 (56, 78)	0.96 (0.22, 1.6)	0.9
**T24**	138 (94, 183)	0.14 (0.06, 0.21)	0.99
**UMUC3**	32 (-3.6, 67)	0.16 (-0.15, 0.47)	0.81

Table 8: VR vs SR linear correlation parameters. The model used was $R(D) = h \times SR(D) + k$. Coefficients are shown with 95% confidence bounds.

Cell line	h	k	R^2
**RT4**	-0.98 (-1.4, -0.61)	0.77 (0.64,0.9)	0.9
**T24**	-0.89 (1.6, 0.12)	0.75 (0.44, 1)	0.64
**UMUC3**	-0.32 (-0.45, -0.2)	0.31 (0.23, 0.38)	0.9

Table 9: Multiple regression model correlating SF, SR and VR for T24 cell line. The model used was: $SF(D) = X_0 + X_1 \times SR(D) + X_2 \times VR(D)$

	Estimate	SE	t Stat	p-value
**X0 (Intercept)**	0.51	0.11	4.4	0.011
**X1**	0.49	0.16	3	0.037
**X2**	-0.005	0.0014	-3.8	0.019

Reference :

Nickerson, M.L., Witte, N., Im, K.M., Turan, S., Owens, C., Misner, K., et al. (2017). Molecular analysis of urothelial cancer cell lines for modeling tumor biology and drug response. *Oncogene* 36(1)**,** 35-46. doi: 10.1038/onc.2016.172.
